# Shaping legal abortion provision in Ghana: using policy theory to understand provider-related obstacles to policy implementation

**DOI:** 10.1186/1478-4505-11-23

**Published:** 2013-07-06

**Authors:** Patience Aniteye, Susannah H Mayhew

**Affiliations:** 1 Department of Community Health, University of Ghana, Legon, Accra, Ghana; 2 Department of Global Health and Development, London School of Hygiene & Tropical Medicine, 15-17 Tavistock Place, London WC1H 9SH, UK

**Keywords:** Abortion, Ghana, Health policy, Lipsky, Policy implementation, Policy theory, Qualitative research

## Abstract

**Background:**

Unsafe abortion is a major public health problem in Ghana; despite its liberal abortion law, access to safe, legal abortion in public health facilities is limited. Theory is often neglected as a tool for providing evidence to inform better practice; in this study we investigated the reasons for poor implementation of the policy in Ghana using Lipsky’s theory of street-level bureaucracy to better understand how providers shape and implement policy and how provider-level barriers might be overcome.

**Methods:**

In-depth interviews were conducted with 43 health professionals of different levels (managers, obstetricians, midwives) at three hospitals in Accra, as well as staff from smaller and private sector facilities. Relevant policy and related documents were also analysed.

**Results:**

Findings confirm that health providers’ views shape provision of safe-abortion services. Most prominently, providers experience conflicts between their religious and moral beliefs about the sanctity of (foetal) life and their duty to provide safe-abortion care. Obstetricians were more exposed to international debates, treaties, and safe-abortion practices and had better awareness of national research on the public health implications of unsafe abortions; these factors tempered their religious views. Midwives were more driven by fundamental religious values condemning abortion as sinful. In addition to personal views and dilemmas, ‘social pressures’ (perceived views of others concerning abortion) and the actions of facility managers affected providers’ decision to (openly) provide abortion services.

In order to achieve a workable balance between these pressures and duties, providers use their ‘discretion’ in deciding if and when to provide abortion services, and develop ‘coping mechanisms’ which impede implementation of abortion policy.

**Conclusions:**

The application of theory confirmed its utility in a lower-middle income setting and expanded its scope by showing that provider values and attitudes (not just resource constraints) modify providers’ implementation of policy; moreover their power of modification is constrained by organisational hierarchies and mid-level managers. We also revealed differing responses of ‘front line workers’ regarding the pressures they face; whilst midwives are seen globally as providers of safe-abortion services, in Ghana the midwife cadre displays more negative attitudes towards them than doctors. These findings allow the identification of recommendations for evidence-based practice.

## Introduction

The prevalence of unsafe abortion in Ghana is a matter of concern; it is thought to contribute to the country’s unexpectedly high maternal mortality rates of 580 per 100,000 live births [1,2]. Complications of unsafe abortion are thought to constitute 22 to 30% of all maternal deaths, thus making unsafe abortion the highest contributor to maternal mortality in Ghana [3]. Since 1985, abortion in Ghana has been permitted in law provided it is carried out by registered medical practitioners in registered facilities and where a pregnancy is the result of rape, incest, its continuation would result in injury to a woman’s physical or mental health, or the foetus has a substantial risk of a serious abnormality [4]. However, the inclusion of this legal clause within the Criminal Code has led to its widespread interpretation as a criminal offence (which it is only if it breaches any of the conditions above). It took twenty years to translate the law into working policy documents within the Ministry of Health (MOH); a three pronged approach to tackle abortion now exists: contraceptive use, provision of safe-abortion services, and the effective management of complications of unsafe abortion are set out in the National Reproductive Health Service Policy and Standards of the MOH and Ghana Health Service [5,6]. This policy document specifies that safe-abortion services are to be provided where permitted by law and abortion complications are to be managed or referred. There is little evidence, however, that safe-abortion services are available in public health institutions [2,7]. Abortion policy in Ghana is currently a particular priority for investigation because of the unexpectedly low impact its effective legalisation has had on reducing the maternal mortality rate in Ghana – the reduction of which remains a priority of the Government of Ghana.

Although it is important to have a policy in place as a first step to achieving access to safe abortion, clearly implementation (and subsequent impact) does not automatically follow. Traditional policy evaluations tended to focus on outcomes and impacts, and when programmes failed to achieve the desired effect the policy design was often blamed. Policy implementation, however, is complex and a good body of literature on theories of policy implementation has developed over the last 35 years, shedding light on multiple influencing factors (for example: [8-16]). These theories can offer possible explanations of the differences in interpretation and practice of laws and policies informing how policy implementation can be better achieved, but such theories are rarely tested [17,18].

With the development of policy analysis and other research techniques (such as realist evaluation), there was an appreciation of the need to explain why and how change did or did not happen, and to look at the role played by actors, processes and the context in shaping change (perhaps the best known references being the study by Walt and Gilson in 1994 [19]). However, the tendency was to focus on actors with substantial and obvious power to effect change. Lipsky’s *Street Level Bureaucrats* theory highlights that frontline health providers do not just mechanically implement the policies that are designed by their superiors [9]. They have discretion with regards to what they implement and how they implement it. They use this power of discretion to develop strategies to deal with the conflict between what the policy demands and what the resources at their disposal allow (Lipsky calls these ‘coping mechanisms’ – referring to how providers balance competing demands). Like many policy theories, Lipsky’s *Street Level Bureaucrats* theory has never really been tested outside the high-income country setting (the United States) where it was developed, though a few studies in sub-Saharan Africa have drawn on it [20,21].

We identified Lipsky’s theory as particularly relevant for looking at the implementation of Ghana’s abortion policy because of its focus on front-line providers. Abortion – and its legal provision by the health sector – evokes high emotions, especially in societies with strong religious or cultural aversion to abortion. Frontline health providers are part of, and influenced by, their cultural and religious context, and their attitudes, values and subsequent behaviour regarding provision of legal abortion services, could be a key barrier to access.

Literature from Ghana highlights the importance of provider attitudes which are often problematic when accessing health services in general [22,23]; a few studies on abortion specifically highlight that negative attitudes of health facility staff impede access to services [7,24]; this is confirmed by other studies from the region [25-30]. Several of these studies also identify the influence of religion and morality on providers’ attitudes to abortion service provision. Nevertheless, there are shortcomings and gaps in the existing literature. No studies from Ghana have systematically sought to understand the role of front-line providers in implementation of the abortion policy or investigate in what ways provider attitudes and practices shape policy implementation. Furthermore, much of the literature on front-line providers in sub-Saharan Africa looks at resource constraints, workload, incentives, or interpersonal relationships. As noted above, the abortion-specific literature indicates the importance of provider attitudes and values in relation to abortion and abortion-services, over and above other systems-related issues. Existing studies have not, however, explicitly examined in what ways provider attitudes and values affect the implementation of abortion policy.

Lipsky’s *Street Level Bureaucrats* theory was developed to explain conflicts providers faced between policy demands and constrained resources. In the present case, it was applied to investigate the highly-charged policy of abortion provision, using its principles of understanding how front-line providers dealt with conflicts and tensions they faced in implementing policies to explicitly identify how provider values and attitudes shape and change the way they implement abortion policies in Ghana. In particular, the theory enabled us to narrow our focus of study and categorise the practices of providers in order to generate recommendations specifically targeted at major, deep-rooted obstacles (provider values and attitudes) to implementation of abortion policy. Furthermore, by applying Lipsky’s concepts to the abortion policy in Ghana we were able to provide a rare test of the theory’s utility in a low-middle income setting and against a sensitive and emotive policy issue.

## Methods

The study was based in Accra, the capital city of Ghana. The study focus was on the implementation of the abortion law and policy and it was considered that people who live in the capital city (i.e., the seat of government) were better placed to be knowledgeable about the research topic.

First, a document analysis of the law, policy documents, and working documents used in the formulation of the policy generated a list of relevant individuals who had been involved in the policy process. These included policy makers, NGO staff, academics, journalists, lawyers, and health professionals of different levels (managers, obstetricians/gynaecologists, midwives) at three large hospitals in Accra as well as staff from smaller and private sector facilities. Second, 76 in-depth interviews were conducted, including 43 health professionals, who were purposively sampled. Interviews were tape-recorded (mostly in English) then transcribed in English. All interviews were conducted by the first author. The topic guide was pre-tested among five doctors and three midwives, then finalised. Data were managed using NUD*IST software and analysed using a framework approach.

### The sample and its context

Data in this article refer to 43 health-care providers (hereafter referred to as health professionals), as shown in Table 1.

**Table 1:** Health professionals interviewed, by facility type

**Group**	**Teaching hospital**	**Regional hospital**	**District hospital**	**Other public sector unit**	**Private facility**	**Pharmacy**
**Obstetrician/ Gynaecologists (n = 15)**	8	3	2		2	-
**Midwives (n = 14)**	8	1	-	4	1	-
**Pharmacists (n = 7)**	7	-	-	-	-	7
**Other health professionals (n = 7)**	5	-	-	2, 1 an NGO	-	-

Accra has one teaching, one regional and one district hospital, as well as a host of urban health centres, privately owned hospitals, clinics and maternity homes where reproductive and child health services (RCH units) are provided. The hospitals have obstetrician/gynaecologists (all obstetricians in Ghana are also gynaecologists; their training equips them to perform the dual function of obstetrician/gynaecologists) and midwives working in their obstetrics and gynaecological units, whilst the RCH units in health centres are staffed by midwives.

Health professionals were purposively sampled from a range of public and private facilities, staff being selected with the help of the unit/facility heads. The three large hospitals in Accra (the teaching, regional and district hospitals) were selected since these see referred cases from across the country and capital city; these workers have substantial knowledge, exposure and experience of abortion. Midwives, rather than nurses, were included because it is they who staff the RCH units providing antenatal, post-natal and family planning services where women in need of reproductive health care services most commonly present. In addition, three private sector establishments were included to give a view of practices in the private sector. The selection of the private practitioners was based on foreknowledge by the first author that one (obstetrician/gynaecologist) provided abortions in his clinic and another (obstetrician/gynaecologists) was a member of an advocacy group for comprehensive abortion care. A group of private midwives were met after one of their general meetings, and on the recommendation of their leader, one member who was willing to be interviewed was included in the study.

In total 15 obstetricians, 14 midwives and 14 ‘other’ health professionals (seven pharmacists, five trainers in medical and midwifery training institutions, a researcher and a representative of an NGO working on women’s reproductive health issues) were interviewed. The 15 obstetrician/gynaecologists had three to four years’ post-graduate training following medical school as well as one to two years’ internship in sub-specialties of their choice. Their ages ranged from 38 to 70 years; all but one of the obstetricians were male. Two of the 15 obstetricians were Moslems and 13 were Christians, four of whom were Catholics. Their working experience ranged between 7 and 30+ years. The vast majority of the obstetricians were of the Akan ethnic group with two northerners and two Ewes. The midwives age ranged from 40 to 60+ years. In Ghana, all midwives are females; one midwife was a Moslem and the remaining were all Christians of the Orthodox and Charismatic category. Their working experience ranged from 12 to over 30 years. Like the obstetricians, they were mostly of the Akan ethnic group. The pharmacists were between 28 and 49 years old; comprising three males and four females. They were all Christians. All were Akan except two who were from the Ga Adangbe ethnic group.

The pharmacists were included in the study because in Ghana studies have shown that community pharmacy shops are the first point of call when women have an unwanted pregnancy since abortion services are not openly available in public hospitals and private clinics are very expensive. Also, some pharmacy shops are known to sell abortifacients (e.g., Cytotec) to women seeking abortion although this is not an over the counter drug. The inclusion of the trainers became necessary since the pilot interviews with obstetricians indicated that controversies existed among trainers regarding provision of abortion services and this, therefore, influenced the teaching of the topic ‘abortion’ to medical or midwifery students. The trainers themselves had all worked as health providers and were now teaching medical students and student midwives. The NGO representative (also originally a trained medical officer who had undergone an internship programme in obstetrics and gynaecology and was involved in training providers in abortion care) was interviewed because of the involvement of the organization in women’s reproductive and sexual health, and more specifically, training and equipping eligible health professionals to provide comprehensive abortion care. Finally, the researcher was included because she worked in a public health institution, had had regular contact with health providers, and was actively involved in many meetings, conferences, and research on abortions. These groups of health professionals were believed to have a wealth of knowledge and experience to share on the topic under investigation and form the essence of sampling for qualitative inquiry.

### The setting: legal and illegal abortion provision in Ghana

Generally, abortion is described as illegal in Ghana unless it is carried out by a medical practitioner in a designated facility following i) rape, ii) incest, and iii) foetal abnormality, or to protect the mother’s physical and mental health [4]. Although most Ghanaians do not know the law, they have the notion that abortion is forbidden.

In Ghana, comprehensive abortion care, which comprises safe-abortion services and post-abortion care, lies within the domain of midwives and obstetricians. The Ghana law on abortion (PNDC Law 102, which is found in the Criminal Code of Ghana) requires that abortions are performed only by medical practitioners (interpreted as doctors). Before 2006, midwives were only eligible to provide post-abortion care whilst the doctors performed both post-abortion care and safe-abortion services. Following the development of the Ghana Health Service Standards and Guidelines for comprehensive abortion care (2006), training of midwives in the provision of safe-abortion services then commenced and currently midwives are reportedly providing comprehensive abortion care.

Private hospitals are known to carry out safe abortions for high fees. However, abortions are not usually openly available in public health facilities for fear of prosecution though they are provided by doctors in a clandestine manner and often labelled as ‘incomplete’ (spontaneous abortions) or diagnostic dilatation and curettage. Some health and paramedical staff, including nurses (especially male nurses), and some doctors also carry out abortions clandestinely in private undesignated premises, but these are against the law and are liable to face prosecution, although prosecutions are few and rare. Ghanaian women who seek abortion may do so from pharmacy shops, private hospitals, some public hospitals and other areas, such as clandestinely in the ‘providers’ homes and usually for high fees. There is not much information on the pathways through which women seek care, but often they will present to a midwife or health professional that they know and trust. They are then either scared away or told that abortion is illegal, counselled against it on religious and moral grounds, or referred to a reproductive health facility, e.g., the family planning clinic, to see a doctor. The woman may or may not get the abortion done at this clinic. The outcome of her referral depends on the doctor’s decision.

### Ethical considerations

Ethical approval for the study was obtained from the ethics review committee of the London School of Hygiene & Tropical Medicine as well as that of the Ghana Health Service. Written permission was also sought from the various heads of institutions from which the sample was drawn.

After contacting interviewees about the project, a time and location was mutually agreed with those who gave their consent to be interviewed. All the respondents were given information sheets with a detailed description of the nature of the study, its purpose, and rationale. The researcher explained the study in detail to all respondents. When they had understood what the study was about and expressed interest in participation; they were asked to sign a consent form. No respondent was coerced to join the study; participation in the study was voluntary. Confidentiality was ensured by selecting locations where the respondent felt comfortable and had audio privacy. Anonymity was ensured in a variety of ways. No names were used in the research report or on the transcripts. All participants were described by their professions and a code – each ‘group’ had at least seven respondents in its pool protecting individual identities from being ascertained. Only the authors had access to the data collected which were kept locked in a cabinet in the researchers’ office.

## Results

We asked respondents to give their views on abortion care (as distinct from abortion, which they also gave) and explored why they did or did not provide these services. A range of arguments emerged which are discussed here, before exploring how these arguments shape health workers’ response to the dilemmas they face and influence their subsequent actions on abortion service provision.

### Provider arguments for and against abortion care

The main arguments put forward in favour of abortion care were on grounds of public health upheld in legal and professional codes. These basically constitute arguments showing the existence of a clear medical problem (evidenced by data) which the professionals saw as warranting their intervention based on ethics of their profession, and the legal and human rights mandates accorded to their profession. The arguments advanced against the provision of comprehensive abortion care were mainly based on religion and morality as well as the rights of the unborn child.

#### Public health

Public health arguments were put forward by 14 obstetricians, all of the ‘other’ health professionals (14) and 11 of the midwives. Several obstetricians noted that if women had no access to safe-abortion services, they would resort to dangerous means of terminating unwanted pregnancies, ending up in hospital which they referred to as ‘sitting back and cleaning up the mess’ or doing ‘post-abortion maintenance’ and contributing to Ghana’s high maternal mortality rate:

*“… about 30% of maternal deaths is due to unsafe abortion … provision of safe-abortion services is one of the easiest ways of reducing maternal mortality.”***Obstetrician 4, age 42**

*“Maternal deaths from abortion is over 30-35% and these are young girls…I don’t have anything against it; so long as that person needs it the person should be referred to the right place.”***Midwife 14, age 60+**

One of the older obstetricians described the lack of abortion services as contrary to international goals:

*“… there are too many women dying from unsafe abortion in our hospitals. We have signed on to the MDGs. We have to reduce maternal mortality […] It is not as if we don’t know what to do. We know perfectly well what to do! So why are they still dying?”***Obstetrician 6, age 70**

Several obstetricians argued that women with unwanted pregnancies suffered mental unrest and would go to all lengths to terminate pregnancy. Obstetrician 13 noted that mental health grounds have been neglected as a reason for abortion on medical grounds:

*“Another example is the emotional aspect. Formerly we did not put much emphasis there. The emphasis was all on physical. It had to be severe hypertension; something that you can physically see on the woman. Now we are giving more meaning to the emotional (mental) aspect. When the woman comes to say that this pregnancy I am carrying was by accident and it is disturbing me mentally …”***Obstetrician 13, age 40**

#### Professional ethics

The majority of obstetricians (12), just over half the midwives (8), and a few of the ‘other’ health professionals (5) referred to professional ethics as a reason for providing abortion care – sometimes qualifying the circumstances. For Ghanaian doctors this is the Hippocratic Oath; for Ghanaian midwives it is the Midwives Prayer and other codes of ethical practice. Even though a clause in the Hippocratic Oath (sworn by newly qualified doctors) forbids abortion, both the Oath and Midwives Prayer enjoin doctors and midwives to ‘save lives’. In the interviews, obstetricians interpreted ‘saving lives’ as meaning the life of already living people (i.e., mothers) and they described a continuum of saving women’s lives in emergency medical situations as well as responding to other physical and mental health needs:

“*… as a practising obstetrician, I sometimes have to make the choice about whether I save this child’s life or the mother’s. There are circumstances in which I cannot save both so I have to take one life in order to allow the other one to live. It is not a happy choice but I have to do it! My upbringing [training] is that I must sacrifice the child to save the mother. That’s the principle …”***Obstetrician 6, age 70**

Obstetrician 13, a younger professional, also acknowledged that obstetric practice demands that they save the mother rather than the foetus and recognised both mental and physical reasons for an abortion. He described abortion as resorting to the ‘greater good’ and argued that saving the mother would ensure the survival of the woman’s other children who depended on her.

The Standards and Protocols of the Ghana Health Service recognises that health providers may claim conscientious objection but states that they are duty-bound to refer women in need of abortion service to an accessible qualified provider. Two midwives (2, 9) reported referring women with unwanted pregnancies to see doctors who could give advice and perform abortions:

*“Unsafe abortion kills and maims women … my profession demands that I save lives, so I refer. I think I have done the best by referring her.”***Midwife 2, age 50+**

#### Human rights

Human rights arguments were put forward both for and against abortion care. Thirteen of the obstetricians, the majority of ‘other’ health professionals (9), but only two midwives cited the human rights of women as a reason to provide abortion care. They argued that based on Ghana’s 1992 Constitution and international conventions such as the International Conference on Population and Development in Cairo (enshrining the right to abortion where legal), which Ghana has adopted, it is the statutory right of women to have abortion services if they need them:

*“It is the right of every woman who wants abortion services, that it should be provided. I want to see a day that women will go to the Ministry of Women and Children’s Affairs holding placards saying that it is their right to have abortion services. Those who talk about the rights of the foetus are bringing religion in.”***Obstetrician 8, age 50**

*“When you look at the Cairo Conference, it was stated categorically that it is the right of the woman to decide … Also, it said where abortion is not against the law, it is the right of the woman to access safe-abortion services so it is a human right … those of us in obstetrics put the right of the woman above that of the foetus …”***Obstetrician 13, age 40**

The two midwives who mentioned human rights were more circumspect than the obstetricians stopping short of saying all women have a right to abortion but introducing a notion of assessed need (women must ‘qualify’):

*“… so if somebody comes and the person qualifies to have an abortion, it is a right. We should not deny them their right …”***Midwife 6, age 60+**

These midwives and some obstetricians also acknowledged the rights of the foetus but the arguments were not clear-cut and highlight the uncertainties and complexities that confront health providers in abortion care provision. A minority of respondents, primarily from the ‘other’ health professionals group, argued strongly against the provision of abortion on the basis of the rights of the foetus:

“*I am not in favour of it [abortion] now and I will never be in favour of it, and wherever I am given the opportunity I will speak against it … Yes, the foetus also has got rights; the right to live.”***Trainer 3, age 54**

#### Religion and morality

The arguments put forward against safe-abortion services were overwhelmingly on the grounds of religion and morality.

Morality was often cited in conjunction with religion making it hard to disentangle the two. From our interviews ‘morality’ seemed to be equated with ‘western, decadent, sexually liberal’ behaviour which is seen as ‘immoral’. Morality was also perceived in terms of arguments about ‘good’ or ‘evil’ more akin to religious beliefs but not given as explicitly religious. Together with religion, ideas about morality were among the major deterrents to the provision of safe abortion.

Many midwives were against abortion and showed scepticism about abortion care and cited religious or moral reasons for rejecting provision of abortion care. The most popular Bible quotation cited against abortion was Jeremiah 1:4–5.

*“… they [abortion providers] are destroying human life. As the Bible says in Jeremiah 1:4-5-‘The word of God came to me saying before I formed you in the womb, I knew you; before you were born I set you apart; I appointed you as a Prophet to the nation’ … the word of God is so clear […] God knew us before we were formed so to destroy … we’re violating the rules of the creator.”***Midwife 12, age 50+**

The command to not destroy life was interpreted only in relation to the foetus, which was often described as a ‘person’ and many midwives expressed an obsessive desire not to contravene this holy writ, such as a midwife who had worked in the gynaecological outpatients’ department of the teaching hospital for over a decade:

“… *I am against abortion because God says keep your bodies as a holy temple for me to come and dwell in you … I wouldn’t want to offend my God. Because he says don’t do it. […] When you do it [abortion], your hands become bloody. The bible tells us that when you do it you are killing. When you destroy that person you have blood on your hands.”***Midwife 8, age 50+**

Two other midwives (Midwives 1 and 5) talked of having ‘bloody hands’ when they get involved in abortion. One of them said her hands are ‘healing hands’ and not ones for ‘killing’.

Although religious arguments were primarily used by opponents of safe abortion (mostly midwives), some respondents who were mainly ‘pro’ abortion also acknowledged religious beliefs and dilemmas. These respondents highlighted the strong religious/moral social context in Ghana and the challenges practitioners (who are themselves inherently religious) encounter:

“*… abortion, sex, Adam and Eve… we are going back to the Bible… people are very uncomfortable about it [abortion]. It doesn’t however diminish the danger to women’s health and lives… I accept that people have a moral problem about abortion. Nobody likes it! […] Pastors shout, preaching dooms day, if you sin God will strike you dead, abortion is bad …. Ghanaians are very religious so the churches have a pervasive influence in which they think that abortion is bad”.***Obstetrician 6, age 70**

A few respondents (obstetricians 2, 9, 13; pharmacist 4) who referred to themselves as religious countered religious opposition to safe abortion citing biblical texts on compassion and forgiveness:

*“Going back to the Bible; adultery is wrong. God had rules about adultery in the Old Testament. A person caught in adultery had to be stoned. But a woman caught in adultery was brought before Jesus Christ and he didn’t have her stoned. This tells me there is more to what you do in a situation. […] I don’t think any of us has the answers… I think a Christian can terminate pregnancy under certain situations.”***Obstetrician 2, age 40+**

*“The way you resolve conflict situations are that you look at the greater good you want to achieve. Jesus Christ also experienced conflict when he had to heal somebody on a Sunday, but he looked at the greater good and went ahead and healed the person […] the greater good is to perform what professionally you have been taught to do!”***Obstetrician 13, age 40**

### Providers’ dilemmas and the complexities shaping implementation of abortion policy

Although the arguments have been presented above as distinct, in reality many respondents advanced two or more reasons why they either supported or opposed comprehensive abortion care. Many respondents displayed tensions between their religious/moral views (mostly about ‘abortion’ itself) and the professional or public health obligations they felt pushed them to provide comprehensive abortion care to those who needed it.

This complex interplay of arguments has an impact on what services health practitioners are willing to provide. This leads to a modification of what is supposed to be provided (as per the standards and protocols of Ghana Health Service) [6] in an attempt to balance the many pressures they face. Drawing on Lipsky’s notions of the use of ‘discretion’ and ‘coping mechanisms’ by street-level bureaucrats we analysed providers’ modifications of the care components of the standards and protocols (counselling, referral, provision of abortion services) that enable them to meet the demands of their profession as well as their deep-rooted religious convictions and socialised views of morality. This allows us to identify where practices could be changed to improve access to safe-abortion services.

#### Counselling

The Ghana Health Service Standards and Protocols state that when a woman reports to a facility for service, the attending health provider should carry out an initial assessment and refer to appropriate services. Pregnancy should be established and the woman asked about what she wants to do. The woman is to be given information on the risks and benefits, all the alternatives (continue pregnancy and parent the child; continue pregnancy and offer child for adoption; terminate the pregnancy where legally permitted) and be supported to make an informed choice. Health providers are cautioned not to impose their beliefs and moral values on clients but to focus solely on client’s needs.

In practice, most obstetricians (11 out of 15) said they offered counselling on all options available to women with unwanted pregnancy to enable them to make an informed choice as required by the standards and protocols. Counselling on all options available for women seeking abortion services appeared to be a ‘routine’ most obstetricians followed. Two obstetricians use their ‘discretionary’ powers during counselling to decide or ‘label’ women with unwanted pregnancies, classifying them as those who ‘genuinely’ need termination of pregnancy and those with ‘flimsy’ excuses for abortion who should carry their pregnancies to term:

“*I will take your history; examine you, if the pregnancy is truly going to be problematic to you, I’ll refer you. If not, and your reasons are just flimsy, I may counsel you to maintain the pregnancy …”***Obstetrician 10, age 40+**

Of the 15 obstetricians interviewed, only two (5, 15) were openly judgemental about women having abortions; for both their Catholic faith was paramount and affected their counselling:

*“… you tell them during counselling that, look here you have committed a crime already. If you go ahead and do this thing [abortion] you are using one sin to cover another sin …”***Obstetrician 5, age 38**

Of the midwives, 13 out of the 14 said they would offer counselling for women seeking terminations (the 14th said she would refer to a doctor without counselling); however, only four said they offered counselling on all the options. Seven only counselled women to deliver the babies:

*“I advise them to keep the pregnancy … I have about 3 or 4 children I call my adopted children […]. At maternity OPD, a woman approached me. She told me she has 6 children and is pregnant again so the husband said she should come to hospital and have an abortion. I spoke to her and invited the husband. I told them, I will help the woman to deliver the baby. After that I will refer them to the family planning people to do sterilization …”***Midwife 8, age 60+**

Midwife 14 also noted with pride that some of the babies born (through her counselling of mothers not to abort) have been named after her, an honour in Ghanaian society. The remaining two midwives said they would only counsel women about the dangers of abortion, over-emphasising the negative outcomes telling women of all the medical, psychological, and religious consequences of abortion. Thus, midwives in particular shaped the content of their counselling as a way of ‘coping’ with the professional need to counsel which conflicts with their religious beliefs that abortion is a sin.

#### Referral to services

Following counselling, women are referred based on the decision taken; to antenatal services if she decides to keep the pregnancy or for safe induced abortion services when she chooses to terminate the pregnancy. Like counselling, referral is the duty of all service providers at all health service delivery levels. While many obstetricians would perform abortions themselves, others preferred to refer:

“… *well, as obstetrician/gynaecologist, I don’t induce abortion but if you come, I will refer you to the family planning clinic [which offers abortion]… I don’t know the outcome because they don’t report back to me […] if you have a genuine problem, I will send you there; if you go and you satisfy the doctors there, okay”.***Obstetrician 10, age 40+**

Even though this obstetrician would refer a woman in need of abortion, he did not want to take the responsibility for the procedure. In using his ‘discretion’ to label women as having a ‘genuine problem’ and that could ‘satisfy the doctors’, he acts as a gatekeeper.

Midwives exhibited a range of attitudes towards referral of women for services mainly based on their explicitly religious beliefs. Although they generally showed conservative attitudes towards abortion care, most (10 out of 14) midwives said they referred women who insisted on seeking abortion services even after counselling. Nevertheless, several (midwives 2, 5, 9) felt uncomfortable referring women for abortion services, believing they are aiding and abetting a ‘sin’:

*“Hm! [gives a big sigh] … in the Christian way, we are not to do abortion, this is a very difficult situation but my work is to prevent death, especially maternal death. So, when someone approaches me with such a situation, I will refer the client to see the doctor … after referring her, I reflect; if it is done for her, it means I have taken part … maybe the doctor will counsel her and will not do it for her.”***Midwife 2, age 50+**

Midwife 5, a Catholic, would not tell women specifically where to go but would only tell them to go to see a ‘qualified doctor’. Midwife 9 prays for forgiveness after referral. Contrary to requirements of the standards and protocols, three midwives said they did not refer women for abortion and one midwife said she engaged the women in conversation concerning their circumstances and then left them to decide what they wanted to do. These actions – mostly an abdication of responsibility through referral – may also be seen as examples of ‘coping mechanisms’ midwives use to withstand the conflicts they experience between their religious beliefs and professional obligations.

#### Provision of services

Twelve out of 15 obstetricians said they provide safe, legal abortion services; however, two of these do so only as a last resort. Two obstetricians (10 and 12) did not provide abortion services themselves but were willing to refer to colleagues who would; one obstetrician (15) said he would neither refer women for services nor provide the service himself.

Despite their willingness to provide safe-abortion services, some obstetricians talked of a stigma of association on doctors who perform abortion. Obstetrician 3 spoke of ‘pressure’ from society on doctors who provide abortions; Obstetrician 2 talked of social labelling:

*“If you are seen to be doing abortions, you are labelled. It will take someone who is strong willed and immune to what people say to offer abortions in a public health facility. Doctors cannot come out openly to speak for provision of safe abortion for fear of being labelled abortionists.”***Obstetrician 2, age 40+**

The interviews showed that it was mainly obstetricians rather than midwives or pharmacists who cited stigma associated with abortion; this is significant. Of the cadres of providers that were interviewed, obstetricians constituted the group that directly provided abortion care and thus were the prime objects of stigma. One way of dealing with stigmatization and social pressures was to conceal the fact that a provider offers abortion services – either by denying it to others or misclassifying it:

*“If you perform abortions why don’t you let people know? They don’t want to be known as abortionists.”***Obstetrician 15, age 66**

*“… sometimes doctors […] they will say ‘diagnostic D and C’ but, you and I know that there is nothing diagnostic about it… in actual fact it’s just an abortion, TOP …”***Trainer 3, age 54**

In fact the abortion standards and protocols demand that there is proper documentation of procedures performed but doctors who perform abortions do not add ‘abortions’ to their operation lists. Only one obstetrician (7) said she openly provides services; she is knowledgeable about the law, works within the framework of the law, documents her cases, and could defend herself.

Importantly, our interviews also revealed that the attitudes of senior health workers in management positions were also critically important in blocking access to safe abortion services – contributing to clandestine provision. These health workers were heads of institutions and in charge of the day-to-day administration of health facilities. They were influential people, often holding prominent positions in the community or religious institutions, and five obstetricians (3,4,6,7,9) most of whom were young and worked under these managers, spoke with frustration of managers’ non-supportive attitudes:

*“… the equipment is under certain ‘authorities’. If the authorities do not believe in making the services available … we are far from the provision … the few people who want to provide the service might not get access to the medical equipment that may be necessary … If my head of department is a Catholic and does not believe in abortions, is he going to provide the right set-up for me to carry out abortions? If I get a complication of abortion I need a senior colleague to take care of the complication. If I am in a set-up where my senior colleague does not believe in carrying out an abortion, where do I go?”***Obstetrician 3, age 38**

Obstetrician 4 said he once approached the head and told him of his plans to provide abortion services in the facility but was told that he did so at his own risk. Others told how managers would not ensure manual vacuum aspiration equipment was procured and that midwives and doctors were discouraged from receiving training:

“*I recollect being told that, ‘I will not allow that nonsense to be performed in my health facility’. This is an administrator of a government hospital so if he says ‘I can’t allow this in my facility’ this was said to prevent people from being trained”.***Obstetrician 7, age 40**

*“… we were discussing with X General Hospital about providing comprehensive abortion services but we had to have the consent of the administrator of the hospital. She initially agreed but when I called she said ‘I have changed my mind because I don’t think I am going to permit this thing to be done in my hospital’ […]. Her personal view should not deny women access to the services in a government hospital!”***Obstetrician 6, age 70**

Thus, many service providers fail to fully implement the standards and protocols on the provision of safe abortion care, choosing to offer services, if they are provided at all, clandestinely rather than openly, impeding access for women, and making them very dependent on highly variable counselling and referral strategies. The general unwillingness to provide services openly can be described as ‘coping mechanisms’ of providers to avoid open association with the provision of socially stigmatised services.

## Discussion

Application and testing of policy or implementation theories is rare in the health services/policy literature, yet theory is an important tool in our quest to promote better understanding of policy and evidence-based service-provision [18]. In this study, we sought to apply the concepts described in Lipsky’s *Street Level Bureaucrats* theory (the dilemmas and pressures faced by providers and their use of discretion and development of coping mechanisms to balance competing demands) to understand how provider attitudes and values shape provider practices regarding safe-abortion service delivery in Ghana and so make recommendations for improving implementation. As far as we are aware, this is the first study to undertake such a systematic analysis of provider attitudes and values towards abortion policy and their role in shaping implementation of services.

We drew on Lipsky’s original concepts of ‘personal dilemmas’ and ‘social pressures’ faced by front-line providers. Lipsky originally conceived these in relation to resources and practice, but as our literature review showed, provider beliefs and attitudes *vis-á-vis* abortion are of critical importance, so we chose to apply Lipsky’s concepts to investigate the dilemmas and pressures that staff beliefs and attitudes create with regards to their professional duty. In our study, the majority of providers experienced religious and moral conflicts in the execution of their professional duties: religious beliefs about the sanctity of life conflicting with their duty to provide abortion care. In addition to these ‘personal dilemmas’ that providers experience, the perceived view of others concerning abortion constituted ‘social pressures’ which affected providers’ decision to (openly) provide abortion services and affected the heads of health institutions who often showed ambivalent and non-supportive attitudes which frustrated their subordinates (obstetricians) and further impeded provision of abortion services. Using the theory’s concepts we examined how providers attempt to achieve a workable balance between these pressures and duties, by using their ‘discretion’ in deciding if and when to provide abortion services, and developing ‘coping mechanisms’. We then identified how these actions affected implementation of abortion policy, specifically counselling, referral, and provision of services.

Our study revealed a number of new findings in relation to how providers’ values and attitudes affect their provision of services and added to existing knowledge about the utility of the *Street Level Bureaucrats* theory for understanding the challenges to implementing sensitive policies in a lower-middle income setting.

### Provider attitudes and values drive modification of policy implementation and vary significantly between staff cadre

Lipsky’s original theory focused on resource constraints as drivers of provider modification of policies during implementation. Our findings show that provider attitudes and values about the policy issue (in this case abortion) are just as important in modifying provider behaviour and thus influencing policy implementation. The theory’s principal mechanisms by which provider actions can be characterised (balancing of pressures through use of discretion and development of coping mechanisms) were shown to be applicable to understanding how providers balanced the personal and professional dilemmas they faced in provision of abortion services.

Analysis of how values and attitudes modified staff implementation of policy showed that different cadres of staff held different views on abortion itself as well as on the provision of abortion-related services, and therefore responded differently to the pressures they faced as they balanced religious beliefs with their professional experiences and exposure to global debates. We found that doctors were generally less judgemental than midwives/mid-level providers and more influenced by their professional judgements than religious or moral arguments. This seemed to be, in large part, because they had much more exposure to international declarations and practice of other (European) countries on provision of safe-abortion services (many had trained in Britain and Eastern Europe and been exposed to safe-abortion service provision and debates) and were consequently more willing to provide services, justifying it on medical, ethical and human rights grounds. They are also, perhaps as a consequence of foreign training, more aware of international conventions and treaties to which Ghana has ratified as well as being more knowledgeable of Ghana’s own law on abortion. They were also more likely to cite Ghanaian public health and medical research showing the detrimental impact of unsafe abortion on maternal health and its contribution to the country’s high maternal mortality rate – a key Millennium Development Goal indicator for Ghana. All these factors act to temper their religious beliefs.

Nevertheless, despite the theoretical willingness of most doctors to provide abortion services, deeper analysis of the interviews revealed that, when confronted with real cases, while counselling referrals are not considered problematic, actual provision of abortion services is usually clandestine. This would seem to reflect a social dichotomy reported in some studies in Ghana that indicate that even though abortion in Ghana is culturally abhorred when exposed, when secrecy is achieved and the outcome of abortion is successful, the procedure is accepted [31,32]. This phenomenon is not unique to Ghana or Africa, being prevalent in Western countries too, and is perhaps a manifestation of the complexities and hypocrisies in the wider discourse on women’s freedom to make choices on their sexual and reproductive rights in which women wishing to exercise their rights are still subject to moral condemnation [33,34].

Midwives are often the first point of contact for a client seeking abortion services; in our study they exhibited the most negative attitudes towards safe-abortion services, counselling women not to abort and refusing to refer them. Many midwives were conscientious objectors on religious grounds often referring to abortion as a ‘sinful act’. Some midwives who did refer clients to providers for help then prayed for forgiveness. The primacy of religious and moral issues in the debate about provision of safe-abortion services is widely documented in the literature [7,26,32,35-37]. Our findings are also confirmed by studies on the religious and moral dilemmas facing nurses in South Africa [25,26] and midwives in France [36]. In Ghana, work by Aboagye et al. [2] who assessed provision of comprehensive abortion care in three regions in Ghana, also found that provider hesitance in providing abortions was because of perceived religious conflicts as well as uncertainty of the legality of abortion, doubts about the standards and protocols for abortion care, and perceived lack of administrative support. There is also a wider literature on the judgemental attitudes of nurses and midwives because of a range of personal, social and structural reasons, which confirm our findings, although these studies are not abortion-specific [20,21,38].

The frequently negative views of nurses and midwives towards provision of safe-abortion services in Ghana – and in other similar settings – calls into question the assumption of some authors (e.g., [39,40]) that allowing mid-level providers to offer abortion services would expand access. On the contrary, our findings suggest that it is obstetricians, with their broadly favourable attitudes, who need to be supported to expand access to care until and unless midwives become more favourable or the availability of misoprostol improves, which could provide an alternative to clinical abortions.

### Power of front-line providers is constrained by mid-level managers, organisational and social hierarchies

Lipsky’s original theory places great emphasis on how providers have a significant degree of power in deciding how a policy should be implemented in practice, thus becoming *de facto* policy makers. We have shown that in fact this personal ‘power’ of front-line providers is (often highly) constrained by organisational hierarchies, including the actions of mid-level managers.

For example, several doctors who wanted to provide safe-abortion services said they had been blocked from doing so openly by their superiors. This partly explains the clandestine practices of doctors described above. The one doctor in our sample who did provide services openly had a good understanding of the legal situation and went to a lot of trouble to document what was done in case of litigation since support from their superiors was not assured.

Opposition came, in particular, from doctors who were heads of health institutions who tended to oppose the open provision of abortion services in institutions they head. They are sometimes accused of failing to support providers who wanted to offer services, preventing them from training, failing to attend meetings where they know the issue of abortion will be discussed. One explanation may be that heads of institutions may hold prominent social positions and therefore feel more socially exposed leading them to block controversial abortion services in facilities that are their responsibility. This stands in contrast to the obstetricians beneath them who more openly expressed favourable views towards provision of safe-abortion services – possibly because they felt more shielded from potential criticism since they were not ultimately responsible for abortion practices at their facilities. Thus, given the multiple competing demands these heads have, they also adapt policy to cope with their particular circumstances (social or professional).

The more judgemental and negative attitudes of midwives towards abortion services may also have to do with the lower hierarchy of midwives in the medical system (as well as in society): doctors are in a position of authority and can make decisions, while lower cadres tend to defer decisions upwards. More than this, though, Lupton’s [41] description of nurses’ position in the medical environment suggests nurses are not well respected by their superiors or the society and experience lower social status and power – something the first author has also experienced first-hand while working in the Ghana Health Service. Building on this, another explanation for midwives’ attitudes and practices may be that religion is seen by midwives as something that gives them moral power over a woman seeking abortion who is seen as having ‘sinned’ and is therefore of a lower social status. Using religion to control ‘sinning’ women’s access to a controversial service is an act of power for midwives that they could not otherwise exercise. Moreover, there was less exposure among midwives to foreign training or global debates and treaties concerning safe-abortion services that seemed to play a role in influencing the more liberal views of obstetricians.

Thus we can see that the *Street Level Bureaucrats* hypothesised by Lipsky do not have complete discretion to modify policy since their choices are in fact constrained by other levels of the system.

### Implications for improving implementation

One of our aims in testing the theory was to assess whether it generated insights that would identify activities that could lead to better implementation of policy. We have shown the critical importance of provider attitudes and values for shaping policy implementation and how these influence their use of discretion and coping mechanisms to modify counselling, referral and provision of abortion services. Our findings indicate that better implementation could be achieved if the negative attitudes of midwives could be tackled and if there was more commitment from mid-level managers to allow legal abortion services in their facilities. A number of possible solutions to this arise. First, the framing of abortion as a ‘health’ (rather than a moral) issue; second, the use of ‘values clarification’; and third, wider advocacy on legal abortion including within the health system.

Although the ‘medicalization’ of abortion has been criticised for perpetuating the domination of doctors in the medical terrain (for example [42,43]), our findings suggest that using a public health framework to ‘medicalize’ the issue could be generally acceptable since it downplays controversial religious and moral dimensions and more respondents seemed sympathetic to medical reasons for abortion (which should systematically be extended to encompass mental health). In particular, public health evidence on the maternal mortality implications of unsafe abortions together with Ghana’s obligations under international treaties (such as the Millennium Development Goals) were frequently discussed by respondents, including those against abortion, as important issues and were mentioned by the obstetricians as arguments that helped to modify their religious position on the issue of safe-abortion. Moreover, our data also show an overarching argument in favour of provision of abortion services on medical grounds – views that cut across all respondent types including those against provision of abortion services in public health facilities. Medicalizing the abortion issue may therefore make it more acceptable and reduce stigma in Ghana, as Lee has documented in the US and others in Nepal [44,45].

Several respondents mentioned the utility of ‘values clarification’ workshops and these could have an important role to play. Values clarification has potential to help transform the attitudes of stakeholders to abortion and abortion care through workshops in which fears and perceptions of abortion are openly discussed and prejudices considered with a view to steering ‘values’ towards a more sympathetic view of the mother’s needs and rights beyond that of the foetus which she carries. Our findings showed that a number of providers judged the clients they saw, considering them to have been somehow at fault for their situation (through adulterous or promiscuous relationships, for example). Value clarification exercises could be incorporated into in-service education programmes and have been successfully used elsewhere [44,46,47].

Our findings indicated a need for advocacy for safe, legal abortion both within the health system, and more broadly in society, if abortion policies are to be implemented to improve access to legal abortion. Klugman and Hlatshwayo [48], in their comparative study of abortion advocacy in eleven countries, note that abortion advocates are frequently more interested in legal change than in following through legal reform with implementation in the public health system. Yet, just as we have shown in Ghana, they note that ‘where service providers are reticent to perform abortions, failure to address this barrier will undermine any other advocacy efforts’ [48: p.39].

In addition to values clarification, the exposure of doctors to international debate on the topic appears to have helped modify their views on safe abortion-provision and promoted open forums for dialogue, discussions and debates among student and practising midwives through organizations such as Nursing/Midwifery Students Associations, the Ghana Registered Nurses Association, and Ghana Registered Midwives Association. This may promote more compassionate and professional responses to women seeking abortion care from midwives. One way to encourage managers to properly implement the MOH policies on safe-abortion services would be to run a series of training sessions on what the policy means and what is possible in law to dispel incorrect notions of illegality. The effect of this, together with regular monitoring visits to institutions tasked with providing safe-abortion services and sanctions implemented where managers refused to attend training sessions or to fully implement the policies, should be tested. A senior member of the MOH who opposed abortion services has now retired and the replacement is much more sympathetic to implementing what is already in law providing a window of opportunity that should be seized to ensure proper implementation.

Changing attitudes, through values clarification and concerted advocacy, is a necessary step, but our findings suggest that proper systems support is also needed. For example, nurses who exhibit unprofessional attitudes towards patients needing any reproductive health service should be sanctioned; regular supervision, support and mentoring of student midwives, practising midwives, and all health providers (in relation to abortion care) by superiors is essential and performance appraisal is also beneficial. All these approaches could be tested in future research.

At a social level, examples from Nepal showed the success of widespread advocacy campaigns and information about where to access safe-abortion services and a national logo was developed that was displayed on all facilities offering the services [49]. Political will and widespread provider support were also key to achieving this in Nepal; however, these are currently lacking in Ghana.

## Conclusions

This study has used a policy implementation theory – Lipsky’s *Street Level Bureaucrats* – to identify what factors shape provision of (legal) safe-abortion services in Ghana. In doing so, we have confirmed that the theory works well in a lower-middle income setting to identify a complex range of influences that providers face and explain their use of discretion and development of coping mechanisms that shape their practice of safe-abortion service provision. Our study has extended the scope and utility of the theory by showing that provider values and attitudes, not just resource constraints, play a critical role in modifying providers’ implementation of policy, especially for morally-charged policies like those on abortion. We also showed that the power of street-level bureaucrats to modify policy implementation is constrained by organisational hierarchies and mid-level managers (to whom the theorised mechanisms of how staff balance pressures to modify policy also apply although managers are not front-line providers). Further, we have established that this theory is a useful tool for identifying how influences manifest themselves differently in different cadres of staff. Understanding these allows us to move towards a more evidenced-based practice by highlighting precisely what needs to be changed for different cadres of staff in order that reproductive health care, including provision of comprehensive abortion care where necessary and legal, becomes more widespread.

While globally there is interest in midwives as providers of safe-abortion services, in Ghana the midwife cadre display more negative attitudes to provision of safe-abortion services than doctors do. Highlighting the public-health evidence of detrimental effects of unsafe abortion, and publically framing abortion as a ‘health’ issue rather than a moral one, could contribute to shaping a more sensitive response among front-line providers in Ghana. Moreover, we have revealed that the MOH must also develop mechanisms to ensure that heads of facilities and departments also properly implement the policies by enabling their obstetricians, who were mostly willing to provide safe-abortion services, to implement these services, where necessary, with proper support.

## Abbreviations

MOH: Ministry of Health; RCH: Reproductive and child health services.

## Competing interests

The authors declare that they have no competing interests.

## Authors’ contributions

PA led the design of the protocol, conducted the fieldwork, led the data analysis, and undertook the full writing of the results (as a PhD thesis). SM advised on the protocol design, and fieldwork, analysed data and wrote this article, based on the PhD thesis. Both authors read and approved the final manuscript.
